# DNA Polymorphisms in Pregnant Women with Sticky Platelet Syndrome

**DOI:** 10.3390/jcm11216532

**Published:** 2022-11-03

**Authors:** Lucia Stančiaková, Jana Žolková, Ľubica Vadelová, Andrea Hornáková, Zuzana Kolková, Martin Vážan, Miroslava Dobrotová, Pavol Hollý, Zuzana Jedináková, Marián Grendár, Tomáš Bolek, Matej Samoš, Kamil Biringer, Ján Danko, Tatiana Burjanivová, Zora Lasabová, Peter Kubisz, Ján Staško

**Affiliations:** 1 National Centre of Haemostasis and Thrombosis, Department of Haematology and Transfusion Medicine, Jessenius Faculty of Medicine in Martin, Comenius University in Bratislava, Martin University Hospital, 036 59 Martin, Slovakia; 2 Centre of Immunology in Martin, s.r.o., 036 01 Martin, Slovakia; 3 Biomedical Centre Martin, Jessenius Faculty of Medicine in Martin, Comenius University in Bratislava, 036 01 Martin, Slovakia; 4 Department of Medical Genetics, Martin University Hospital, 036 59 Martin, Slovakia; 5 Centre of Haemostasis and Thrombosis, Unilabs Slovakia, s.r.o., 036 01 Martin, Slovakia; 6 Laboratory of Bioinformatics and Biostatistics, Biomedical Centre Martin, Jessenius Faculty of Medicine in Martin, Comenius University in Bratislava, 036 01 Martin, Slovakia; 7 Laboratory of Theoretical Methods, Institute of Measurement Science, Slovak Academy of Sciences, 841 04 Karlova Ves, Slovakia; 8 Department of Internal Medicine I., Jessenius Faculty of Medicine in Martin, Comenius University in Bratislava, Martin University Hospital, 036 59 Martin, Slovakia; 9 Department of Gynaecology and Obstetrics, Jessenius Faculty of Medicine in Martin, Comenius University in Bratislava, Martin University Hospital, 036 59 Martin, Slovakia

**Keywords:** sticky platelet syndrome, DNA analysis, polymorphisms, antithrombotic treatment

## Abstract

Sticky platelet syndrome (SPS) is a thrombophilia caused by the increased aggregability of platelets in response to the addition of low concentrations of epinephrine (EPI) and/or adenosine diphosphate (ADP). Some of the single nucleotide polymorphisms (SNP), alleles and haplotypes of platelet glycoprotein receptors were proved to have a role in the etiology of thrombotic episodes When comparing SPS and the control group, in VEGFA rs3025039, the *p* value for both CC vs. TT and CT vs. TT analyses was <0.001. Interestingly, no minor TT genotype was present in the SPS group, suggesting the thrombotic pathogenesis of recurrent spontaneous abortions (RSA) in these patients. Moreover, we found a significant difference in the presence of AT containing a risky A allele and TT genotype of ALPP rs13026692 (*p* = 0.034) in SPS patients when compared with the controls. Additionally, we detected a decreased frequency of the GG (CC) genotype of FOXP3 rs3761548 in patients with SPS and RSA when compared with the control group (*p* value for the CC (GG) vs. AA (TT) 0.021). This might indicate an evolutionary protective mechanism of the A (T) allele in the SPS group against thrombotic complications in pregnancy. These results can be used for antithrombotic management in such pregnant patients.

## 1. Introduction

Sticky platelet syndrome (SPS) represents an autosomal dominant platelet function disorder associated with platelet hyperaggregability in platelet-rich plasma (PRP) with adenosine diphosphate (ADP) and/or epinephrine (EPI). Increased aggregability after the addition of both of these substances is defined as SPS type I, hyperaggregability after EPI alone as type II and increased aggregability only after the addition of ADP is SPS type III [1].

SPS can manifest as arterial thrombosis, such as acute myocardial infarction, angina pectoris, transient cerebral ischemic attack, stroke, peripheral arterial thrombosis, retinal thrombosis, or venous thromboembolism—frequently recurrent despite anticoagulant therapy or pregnancy complications (e.g., fetal growth retardation and fetal loss) [1,2,3,4,5]. Moreover, it has been reported that women with SPS have significantly more spontaneous abortions than patients in the general population [6].

Several mutations of genes encoding platelet glycoprotein receptors and further proteins associated with platelet function have been studied as potential etiopathogenetic factors of recurrent pregnancy loss (RPL) in women with SPS.

Single nucleotide polymorphisms (SNPs) rs9550270 and rs7400002 of the *GAS6* gene responsible for the function of alpha2-adrenergic and ADP receptors and activating endothelial and vascular smooth muscle cells are more common in women with SPS and pregnancy loss [7,8].

Moreover, SNPs 1,671,153, 1,613,662 and 1,654,419 of *GP6* as the gene encoding the receptor for collagen are more frequent in women with SPS and pregnancy loss. A significantly increased occurrence of CTGAG in haplotype 5 and CGATAG in haplotype 6, an increased presence of SNPs rs1671152, rs1654433, rs1654416, rs2304167 and rs1671215 in patients with platelet hyperaggregability and previous pregnancy loss and a significantly higher frequency of ccgt in *GP6*_3reg haplotype, acgg and aagg in *GP6*_5reg haplotype, SKTH and PEAN in *GP6*_PEAN haplotype and gg and ta in *GP6*_REG haplotype in this population have been confirmed [7,9,10,11,12,13].

Patients with SPS and spontaneous abortion had an increased prevalence of SNPs rs12566888 and rs12041331 of the *PEAR1* gene responsible for platelet contact [8].

Increased expression of platelet microRNA (miR-96) is expressed in patients with SPS and pregnancy complications [14]. Conclusively, different mutations of one or more genes might lead to a similar SPS phenotype. Additionally, platelets of individuals with atherosclerosis, renal and autoimmune diseases have hyperaggregability after EPI or other agonists, highlighting the possible existence of acquired forms of SPS [2,7].

In spite of several studies investigating the role of platelet glycoproteins in the activation and aggregation of platelets, the exact underlying defect causing the syndrome has not been fully elucidated [15].

In most patients, low doses of antiplatelet agents (usually 80–100 mg of acetylsalicylic acid (ASA) per day) lead to normalization of platelet hyperaggregability [15] and improvement of pregnancy outcome in comparison with SPS patients without such treatment [16]. However, in risky situations, such as a history of thromboembolic episodes or the presence of prothrombotic changes in hemostasis associated with RPL, both low-molecular-weight heparin (LMWH) and ASA are recommended, as also indicated by Bick and Hoppensteadt [17]. Therefore, pregnant patients in our study used a combination of ASA and LMWH to prevent further complications.

The term ‘recurrent pregnancy loss’ (RPL) is recommended for the description of repeated pregnancy demise and recurrent miscarriage (recurrent spontaneous abortion, RSA) when all pregnancy losses are confirmed as intrauterine miscarriages by histology or ultrasound [18,19]. A pregnancy loss is a spontaneous pregnancy demise before the fetus reaches viability—i.e., until 24 gestational weeks [20].

There is also a variation in the quantity defining recurrent miscarriage. It ranges from two miscarriages reported by the European Society of Human Reproduction and Embryology and the American Society for Reproductive Medicine to three subsequent pregnancy losses, as defined by the Royal College of Obstetricians and Gynaecologists [21].

In general, RPL affects approximately 2–5% of couples. Frequent causes are uterine anomalies, hormonal and metabolic disorders, antiphospholipid syndrome and genetic abnormalities. Further etiological factors that have been investigated include inherited thrombophilia, luteal phase deficiency, chronic endometritis and high sperm DNA fragmentation level [22]. However, it has been proved that approximately 55% of recurrent miscarriages are due to prothrombotic defects inducing infarction and thrombosis of placental vessels [23].

The *vascular endothelial growth factor A* (*VEGF-A*) gene encompasses 14 kb and is localized on the human chromosome 6, consisting of eight exons [24]. It is a member of the platelet-derived growth factor (PDGF)/vascular endothelial growth factor (VEGF) family. *VEGFA* encodes a heparin-binding protein inducing proliferation and migration of vascular endothelial cells. It is thus critical for physiological and pathological angiogenesis [25]. Additionally, *VEGFA* is essential for embryonic vasculature development, stimulation of trophoblast proliferation and both fetal and maternal blood cell growth in the course of early pregnancy. *VEGF* in general is also important for the implantation of the embryo into the placental wall, so its genetic defects have been studied in association with RPL [24]. A decrease in *VEGF* expression in first-trimester tissues can even indicate its involvement in RPL [26].

The *alkaline phosphatase, placental (ALPP)* gene encodes an alkaline phosphatase, a metalloenzyme catalyzing the hydrolysis of phosphoric acid monoesters. One of its main sources is the liver. However, in pregnant women, it is primarily expressed in placental and endometrial tissue. Strong ectopic expression of *ALPP* has been confirmed in ovarian adenocarcinoma, serous cystadenocarcinoma and further ovarian cancer cells [27].

*Fork head box protein 3 (FOXP3)* is an X-linked gene that codes a master transcription regulatory protein controlling the development and function of immunosuppressive T regulatory cells. These cells are key mediators of maternal fetal tolerance [28]. A decrease in T regulatory cells in peripheral blood and decidua leads to a decrease in *FOXP3* gene expression, which affects the development and function of CD4+ CD25+ T regulatory cells [29,30]. The protein encoded by the *FOXP3* gene represents a member of the fork head/winged-helix family of transcriptional regulators. Diseases associated with *FOXP3* include polyendocrinopathy, immunodysregulation, X-linked enteropathy and nonimmune and X-linked hydrops fetalis [31].

Based on this knowledge, the authors aimed to investigate the relationship between SPS, recurrent spontaneous abortions (RSA) and further thromboembolic complications and selected polymorphisms rs3025039 in *VEGFA*, rs2010963 in *VEGF*, rs13026692 in *ALPP* and rs3761548 in *FOXP3* genes.

## 2. Materials and Methods

### 2.1. Patients and the Control Group

A total of 53 pregnant women of Caucasian origin with a sticky platelet syndrome, 21 pregnant patients with a history of unprovoked or estrogen-related thromboembolic complications and 53 pregnant women with a history of RSA receiving antithrombotic thromboprophylaxis were included in the study.

SPS was diagnosed in patients before their inclusion in the study via light transmission aggregometry with the analysis of responsiveness of platelet-rich plasma to three different concentrations of adenosine diphosphate (ADP) and epinephrine (EPI) according to the criteria of Mammen and Bick [7] (Table 1). We suspect this diagnosis when the patient has a history of thromboembolic episodes and proved platelet hyperaggregability after mixing of the sample with 1 concentration of 1 of these reagents. The diagnosis of SPS is confirmed when the patient has one of the combinations of these situations:

- A history of thromboembolic episodes and hyperaggregability after the use of 2 concentrations of 1 reagent;
- A history of thromboembolic episodes and hyperaggregability after the use of 1 concentration of both reagents (ADP and EPI);
- A history of thromboembolic episodes and hyperaggregability after the use of 1 concentration of 1 reagent, but repeatedly tested [7].

As mentioned above, the form of primary thromboprophylaxis in SPS is the use of ASA; however, in the case of the development of prothrombotic changes in hemostasis during pregnancy (e.g., significantly increased FVIII activity or decrease in free PS), combined antithrombotic prophylaxis composed of ASA and LMWH had to be used.

Due to the increased risk of bleeding during the use of such prophylaxis, pregnant patients with the following clinical conditions predisposing to bleeding were excluded from the study: a history of hemorrhagic stroke, disorder of blood coagulation or other diseases contributing to bleeding (severe thrombocytopenia, history of thrombocytopenia developed after the use of anticoagulant drugs, active gastroduodenal ulcerations, severe renal insufficiency (creatinine clearance <30 mL/min.), acute infective endocarditis and a history of severe allergic reaction to antithrombotics).

RSA was confirmed by a gynecologist with the exclusion of further causes of this complication, such as anatomic, hormonal or genetic changes or infections. Mean age was 31.93 years (age range 19–46 years), and the number of RSA varied from 2 to 8. Inclusion of patients was carried out from January 2014 to March 2019.

During clinical examination, data about family and personal history, drugs, allergies and gynecological history (previous abortions, interruptions, deliveries or thromboembolic complications) were collected.

The control group comprised 58 healthy non-pregnant women without any personal or family history of thromboembolism and no history of pregnancy complications, such as placental abruption, RPL in general, fetal demise, intrauterine growth restriction (IUGR) or VTE during pregnancy. These subjects did not take any agents that could have an impact on hemostasis—anticoagulant drugs, antiplatelet agents or oral contraceptives. The mean age was 29.05 years (age range 18–45 years).

We compared the frequency of genotypes of particular SNPs between four groups—the results of pregnant women with SPS (designated S in the figures and tables), of those with a history of RSA (group A in the figures and tables), of those with a history of thromboembolism (T) and of the control group (C).

### 2.2. Processing of Blood Samples for Genotyping

For genotyping, 10 mL of antecubital venous blood was obtained from each fasting pregnant woman included in the study and each fasting woman from the control group.

Blood was collected in Vacutainer^®^ blood collection tubes with ethylenediaminetetraacetic acid (EDTA) as an anticoagulant, then immediately stored at 4 °C and further processed within 6 h. Centrifuging of the blood samples was carried out at 3000 rpm at 4 °C for 10 min to separate the serum plasma and buffy coat containing white blood cells, and then frozen at −20 °C for DNA extraction and genotyping.

Genomic DNA was isolated from buffy coat using a DNeasy Blood and Tissue Kit (Qiagen, Germany). All DNA samples were diluted to 20 ng per μL and were used as a template for genotyping.

The AB 7500 Fast Real-Time PCR system (Applied Biosystems, USA) was used to analyze polymorphisms rs3025039 in *VEGFA* (assay ID: C__16198794_10), rs2010963 in *VEGF* (assay ID: C__8311614_10), rs13026692 in *ALPP* (assay ID: C__11531497_10) and rs3761548 in *FOXP3* (assay ID: C__27476877_10). Each TaqMan genotyping assay mix contained a forward and reverse primer, one probe with perfect matching to the wild-type sequence variant labeled with VIC and the other probe labeled with FAM with perfect matching to the mutant sequence variant. TaqMan allelic discrimination real-time PCR was performed in a 20 μL volume, containing 0.5 μL TaqMan genotyping assay mix, 10 μL TaqMan Genotyping Master Mix (Applied Biosystems, Waltham, MA, USA), 7.5 μL DNase-free water and 2 μL of diluted genomic DNA. The real-time PCR conditions were as follows: an initial step at 95 °C for 10 min, followed by 50 cycles of denaturation at 92 °C for 15 s and annealing/extending at 60 °C for 1 min and 30 s. The genotypes were detected according to the strength of the fluorescent signals from VIC/FAM labeled probes.

### 2.3. Statistical Analysis

The role of this study was to explore how exactly the selected SNPs can predict the probability of the tested person belonging to one of the following groups: SPS/RSA/control group/thromboembolism. Therefore, we used multinomial logistic regression analysis, and the result was expressed as the significance of particular alleles of all SNPs and odds ratio (OR). The response was the group, and the predictors were all four SNPs.

For each of the SNPs, we made a contingency table showing the relationship genotype vs. study group. To obtain a summary contingency table, we performed a Chi-squared test and G-test of independence between genotype and study group. Cramér V was used for an effect size measurement in the contingency table. In cases where H0 was refused for any of the SNPs, we carried out pair post hoc tests (pair comparisons of particular levels of factor in the groups). A *p* value < 0.05 was considered statistically significant. We also adjusted the *p* value based on Holm’s method and the Bonferroni correction.

Moreover, we calculated the estimated marginal means of frequencies of the alleles for each SNP and each group.

The control group was taken as the reference level in the group analysis. In each SNP, the minor allele was taken as the reference.

Not all pregnant women included in the study were treated with ASA or LMWH uniformly, so we performed a multivariate analysis to exclude the effect of antiplatelet drugs/anticoagulants on pregnancy outcomes or the occurrence of thromboembolism as potential confounding factors. For the same reason, we also analyzed the effect of the presence of concomitant thrombophilia in our pregnant patients as another confounding factor.

Statistical analysis was performed using the jamovi project, version 2.3, and the data were explored and analyzed in R (R), version 4.1 [32,33,34,35].

## 3. Results

### 3.1. Clinical Data

Family history in the form of thromboembolic and pregnancy complications (preeclampsia, RPL in general or intrauterine fetal death) was positive in 15 cases. SPS type I was detected in 16 patients and type II in 37 women; we did not include any pregnant woman with SPS type III. The most common dose of ASA used on patients was 100 mg taken daily (60%), while the minimal dosage confirmed as effective before the initiation of the study and used by patients was 50 mg (taken by 16.67%). The maximal dose of ASA was 150 mg daily for one woman.

Two patients with SPS were directly allergic to ASA and thus used only LMWH, while 29.13% of all included patients reported allergic reactions in the form of redness, resistances and local irritation of the skin at the site of administration of LMWH. For this reason, they switched between LMWH products, usually from nadroparin to enoxaparin.

In addition to SPS detected in the 53 mentioned patients, further thrombophilic states diagnosed in at-risk pregnant women were: antithrombin deficiency (*n* = 5), hyperhomocysteinemia (*n* = 8), factor V Leiden mutation present in the homozygous form (*n* = 2), heterozygous form (*n* = 17), prothrombin variant G20210A in the heterozygous form (*n* = 7), heterozygous form of mutation of βFbgc.−39–424 G > A (*n* = 24), homozygous form (*n* = 2), PAI 4G/5G homozygous (*n* = 7) and heterozygous form (*n* = 7), mutation FXI c.1481-188 C > T (*n* = 4), SNP FXI rs2289252 (*n* = 2), variant FXII C46T in the homozygous form present in 2 patients and in heterozygous women (*n* = 1), CYP4V2 homozygous form of mutation (*n* = 3), homozygous form of mutation FXIII Val34Leu (*n* = 1) and the presence of antiphospholipid antibodies (*n* = 6).

No renal or liver function impairment developed. None of the included pregnant patients developed HELLP syndrome or heparin-induced thrombocytopenia. During the study, we did not detect any thromboembolic episode in the included patients.

The control group was composed of healthy non-pregnant women (mean age 29.42 years, age range 18–45 years). Based on the anamnestic data, none of them were pregnant or in menopause during the study.

### 3.2. Results of Genotyping

**Figure 1:**
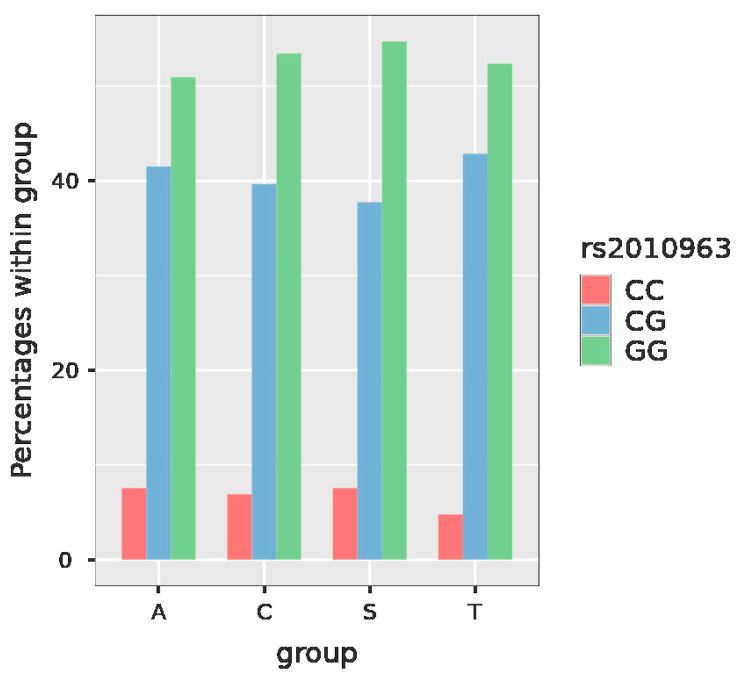
Plot with the frequency of the particular genotypes of *VEGFA* rs2010963 in the studied groups. Legend: group A—recurrent spontaneous abortions, group C—controls, group S—sticky platelet syndrome, group T—thromboembolism, VEGFA—vascular endothelial growth factor A.

In the case of *VEGFA* rs2010963, the possible genotypes are GG, GC and CC. For *VEGFA* rs2010963 in our studied population, the global frequency of the GG genotype was 53%, while that of GC was 40% and that of CC 7% (Figure 1, Table 2).

**Figure 2:**
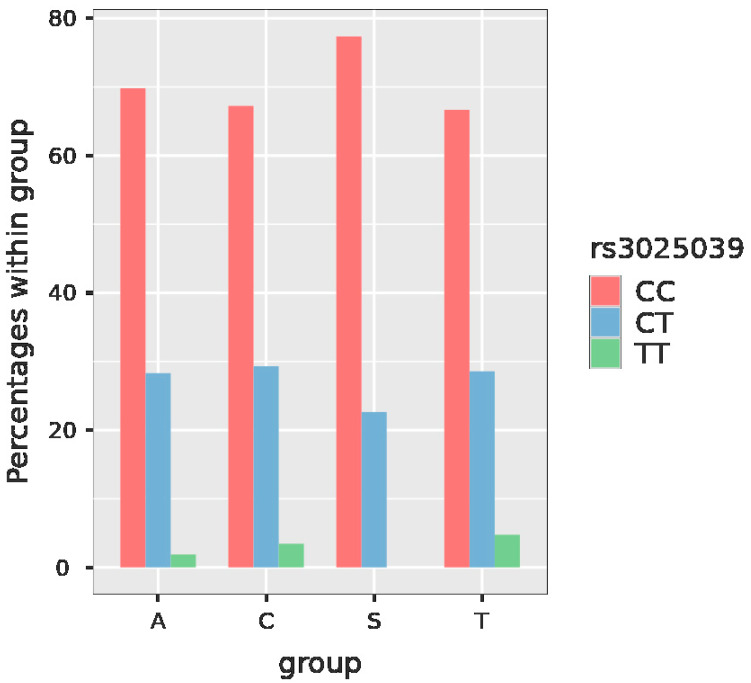
Plot with the frequency of the particular genotypes of *VEGFA* rs3025039 in the studied groups. Legend: group A—recurrent spontaneous abortions, group C—controls, group S—sticky platelet syndrome, group T—thromboembolism, VEGFA—vascular endothelial growth factor A.

*VEGFA* rs3025039 has the possible genotypes CC, CT and TT. The general frequency of the CC genotype in SNP *VEGFA* rs3025039 was 70.8%, the CT genotype was present in 27% of the women included in the study and the TT genotype was detected only in 2.2% (Figure 2, Table 3).

**Figure 3:**
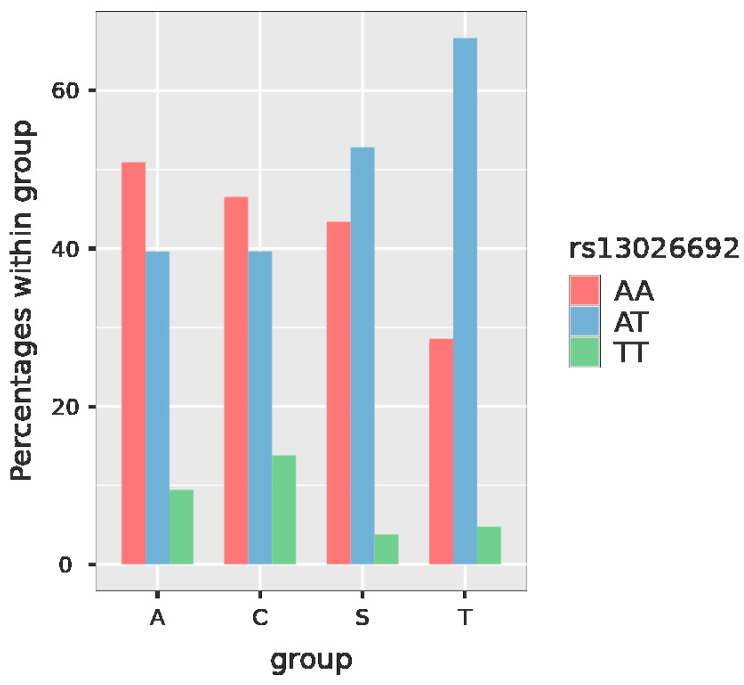
Plot with the frequency of the particular genotypes of *ALPP* rs13026692 in the studied groups. Legend: *ALPP*—alkaline phosphatase, placental, group A—recurrent spontaneous abortions, group C—controls, group S—sticky platelet syndrome, group T—thromboembolism.

*ALPP* rs13026692 has the possible genotypes AA, AT and TT. In the case of this polymorphism in our study, the frequency of the AA genotype was 44.9%, AT was present in 46.5% and TT only in 8.6% (Figure 3, Table 4).

**Figure 4:**
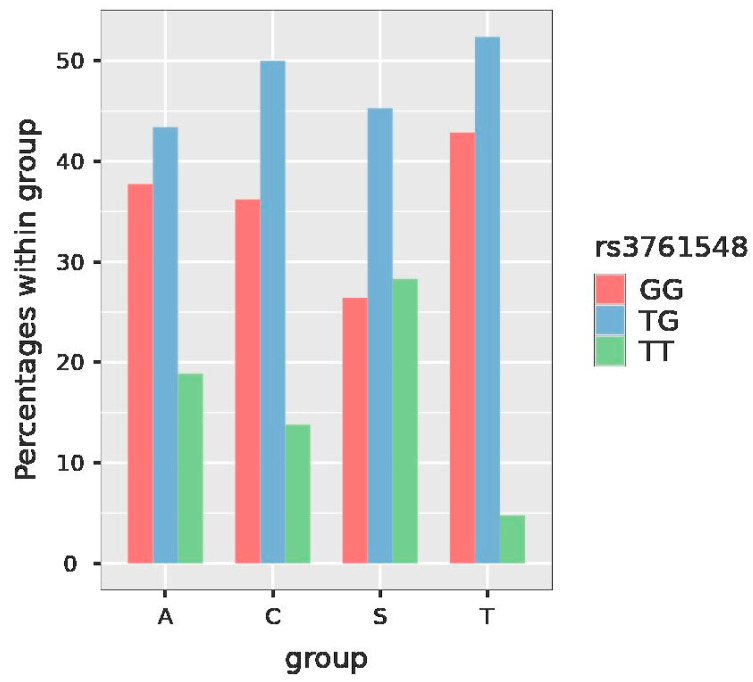
Plot with the frequency of the particular genotypes of *FOX3* rs3761548 in the studied groups. Legend: FOX 3—fork head box protein 3, group A—recurrent spontaneous abortions, group C—controls, group S—sticky platelet syndrome, group T—thromboembolism.

SNP *FOX3* rs3761548 has the possible genotypes CC, CA and AA. For SNP *FOXP3* rs3761548 in our included women, the GG genotype was detected in 34.6% of the women, GT in 47% and TT in 18.4% (Figure 4, Table 5).

Using multinomial logistic regression—group vs. SNPs—when taking into consideration the comparison of the SPS and the control group, in *VEGFA* rs3025039, both CC vs. TT and CT vs. TT analyses showed significant results (*p* value for both of them was < 0.001) (Table 6).

For SNP *ALPP* rs13026692, the comparison between genotypes AT and TT was significant (*p* = 0.034) as well. For SNP *FOXP3* rs3761548, GG vs. TT analysis also showed a significant value (*p* = 0.026). Thus, subjects with the GG genotype are at a four times lower risk of having SPS than subjects with the TT genotype (OR = 0.27). However, the decrease in risk is estimated with a low precision—the 95% confidence interval (95%CI) for odds ratio (OR) was (0.08, 0.86).

In the other group comparisons, we did not obtain significant data.

According to the results of estimated marginal means (estimates of the probability of the particular allele), for SNP *FOXP3* rs3761548, the TT (AA) genotype in the group of the patients with thromboembolism has a significant probability of presence (*p* value = 0.0439).

Using post hoc tests, when analyzing *VEGFA* rs3025039 in the SPS group, the comparisons of the occurrence of genotypes CC vs. TT and CT vs. TT were statistically significant (*p* values < 0.001 and 0.002, respectively) (Table 7).

In the case of *ALPP* rs13026692, the comparison between AT and TT genotype in the SPS group was also significant (*p* = 0.022) (Table 8).

Similarly, for SNP *FOXP3* rs3761548, in the SPS study group, the comparison between GG and TT genotype was evaluated as statistically significant (*p* = 0.021) (Table 9).

In the case of *VEGFA* rs2010963, there were not any significant results between the probability of the presence of two studied genotypes. Moreover, the *p* value in the Chi-squared test for this SNP was 0.999.

However, R^2^McF was 0.0469—this generally indicates a poor prediction ability of the studied SNPs.

When investigating the association between thromboembolism/recurrent spontaneous abortions and SNP, the *p* value for *VEGFA* rs2010963 polymorphism was 0.7486, and Pearson’s Chi-squared test (X-squared) was 0.57917. For *VEGFA* rs3025039, the *p* value was 0.69, and Pearson’s Chi-squared test with Yates’ continuity correction (X-squared) was 0.15906. Regarding SNP *ALPP* rs13026692, the *p* value reached 0.47, and Pearson’s Chi-squared test (X-squared) was 1.51. For SNP *FOXP3* rs3761548, *p* was 0.233, and Pearson’s Chi-squared test (X-squared) was 2.9136.

A multivariate analysis to evaluate the effect of ASA and LMWH on pregnancy outcome in terms of RSA or on the presence of thromboembolism is outlined in Table 10. The effect of concomitant thrombophilic state on the data obtained in the study is assessed in Table 10. In Table 10, we also tested the influence of the age of the patients on the results. Last but not least, post hoc comparisons for particular genotypes of selected SNPs in our study are provided in Table 11, Table 12, Table 13 and Table 14.

Post hoc comparisons of ASA vs. LMWH and those taking into account the influence of other thrombophilia are outlined in Table 15 and Table 16. 

## 4. Discussion

It was confirmed that particularly rs1570360 (−1154G/A) (OR 1.51 (95%CI 1.13–2.03)), rs3025020 (−583C > T), rs833061 (460T/C), rs2010963 (−634G/C) and rs3025039 (+ 936C/T) *VEGF* genetic polymorphisms increase the probability of RSA or RPL [36,37,38,39,40,41]. The last two mentioned SNPs are even associated with an increased risk of preeclampsia in various ethnic groups [42].

In the case of SNP *VEGF* rs1570360 (−1154G > A), the variant allele A was significantly more common in patients with RPL (0.41) than in controls (0.19) (*p* < 0.0001). In *VEGF*-583 C > T, the CT genotype was significantly associated with this pathological state (*p* = 0.003) [43].

RPL is frequent in the population with *VEGF*-1154G/A (70.04%) and p53 Arg72Pro polymorphism (66.46%). The homozygous recessive genotype of *VEGF* and p53 thus exhibits significant association between these polymorphisms and RPL [44].

In *VEGF* 634 G > C, the allele C and CC genotype are significantly more frequent in individuals with RPL than in the control group (*p* < 0.0001) [43]. Thus, the frequency of idiopathic RSA can be dependent on the GC and CC genotype of rs2010963 *VEGF* polymorphism [45].

Moreover, placental −634 GC and CC genotypes might be involved in the development of preeclampsia and also in its severe form [46], with OR 1.85 (95%CI 1.25–2.75) and OR 1.90 (95%CI 1.28–2.83) in the maternal and fetal dominant model [47].

The C allele of SNP rs3025039 is associated with an increased risk of preeclampsia, and the T allele seems to have the opposite effect [48]. Interestingly, based on the results of the meta-analysis of 24 studies, rs2010963 polymorphism significantly contributes to the development of hypertensive disorders of pregnancy in the Caucasian and African population and rs3025039 in Asian women [49].

In our studied population, the GG genotype of *VEGFA* rs2010963 was most commonly found in the SPS group (54.7%). The less risky - minor CC genotype was more frequent in the group of pregnant patients with SPS and in the women with a history of RSA (7.5% in both of them) than in the group with a history of thromboembolism and the control group (4.8% and 6.9%, respectively). However, the *p* value in the Chi-squared test for this SNP was 0.999. This means the absence of a significant relationship between *VEGFA* rs2010963 and the study group and, thus, a poor predictive value.

The CC genotype of the SNP *VEGFA* rs3025039 was detected most commonly in the SPS group (77.4%). When compared with the controls, this was proved to be statistically significant (*p* value for the comparison of CC vs. TT genotype < 0.001), as outlined in Table 6 and Table 7. By contrast, interestingly, the minor TT genotype was not present in the SPS group. This finding confirms an increased frequency of the major (risky) genotype in the SPS population and suggests the thrombotic pathogenesis of RSA in this group of patients.

The T/T (Leu/leu) genotype of *ALPP* showed a protective effect for in vitro fertilization (IVF) failure and primary RSA (RR 0.438 (0.232–0.828, *p* 0.002) and RR 0.532 (0.291–0.974, *p* 0.016)). In the case of secondary RSA, the heterozygous genotype may be a risk factor with an RR of 2.226 (1.383–3.583, *p =* 0.0031) [50].

Our study confirmed an increased frequency of the protective TT genotype in the control group (13.8%) and its lower incidence in the group of patients with SPS and a history of RSA (3.8%). These results were proven to be statistically significant (*p* value for the comparison of AT vs. TT genotype in the SPS group was 0.022) (Table 8). Moreover, for the SPS vs. control group in the multinomial logistic regression analysis, when comparing AT and TT genotype, the *p* value was 0.034 (Table 6). Such findings also correlate with an increased frequency of the risky AA genotype in the group of recurrent spontaneous abortions (50.9%) when compared with the controls (46.6%).

*FOXP3* rs3761548 polymorphism (−3279 C > A) is associated with a reduced expression of full-length FOXP3 protein in patients with unexplained RSA [28], and rs3761548 A/C polymorphism might be a significant risk factor for RPL [51,52]. Additionally, a potential relationship between further variants of *FOXP3* rs5902434, rs2232365 and rs2294021 and idiopathic recurrent miscarriage was confirmed [52,53].

Wu et al. suppose that functional polymorphisms of the *Foxp3* gene can represent an important factor of unexplained RSA in Chinese Han women, probably by altering Foxp3 expression and/or its function [52].

In addition to this relationship, *FOXP3* rs3761548 polymorphism was also tested for its association with preeclampsia. However, this causal link was not confirmed by Varshini et al. [54]. On the other hand, it was suggested that the A allele of this polymorphism might be protective against preeclampsia, and the C allele predisposes to this clinical condition in a dose-dependent manner [55].

We detected a decreased frequency of the GG (CC) genotype of *FOXP3* rs3761548 polymorphism in our study group of patients with SPS and RSA when compared with the control group (*p* value for the CC (GG) vs. AA (TT) genotype in these two study groups = 0.021) (Table 9). This may indicate an evolutionary protective mechanism of the occurrence of the A (T) allele in the SPS group providing protection against thrombotic complications associated with pregnancy (preeclampsia or RSA).

Using a generalized linear model for logistic regression for the assessment of age as a potential factor, the *p* value of the likelihood ratio test was 0.728, whereas in the case of consideration of treatment as a potential confounding factor, it was <0.001. When taking into consideration the presence of other thrombophilia, the *p* value was 0.927, so the addition of this predictor to logistic regression does not improve the prediction regardless of whether the particular patient might be included in the group of thromboembolism or RSA (Table 10).

Thus, age does not have a significant influence on the results of our study. Moreover, after performance of post hoc tests, we did not find any significant difference between the genotypes of particular SNPs analyzed in our study (Table 11, Table 12, Table 13 and Table 14). Regarding the influence of treatment with ASA or LMWH and the impact of the presence of concomitant thrombophilia on our results, we did not obtain any significant data, either (Table 15 and Table 16).

However, when looking at the data of rs3761548, the comparison of the GG and TT genotype is close to statistical significance before the correction for multiple testing (*p* value = 0.114). Therefore, patients with the GG (CC) genotype are approximately 11 times more at risk of thromboembolism than those with the TT (AA) genotype. This correlates with the above-described increased risk of RSA and RPL in carriers of A/C polymorphism and the increased risk of preeclampsia in the carriers of the C allele, as all these clinical states (RSA, RPL and preeclampsia) might be developed on the basis of thrombosis or vascular impairment in uteroplacental circulation. These results need to be confirmed using data from a higher number of patients, so we will continue to include further at-risk pregnant women to confirm our presumptions.

## 5. Conclusions

Our study confirmed the most frequent occurrence of the risky CC genotype of *VEGFA* polymorphism rs3025039, particularly in SPS patients (*p* value < 0.001), in comparison with the TT genotype and the control group. Moreover, we found a significant difference in the presence of AT containing the risky A allele and TT genotype of *ALPP* rs13026692 polymorphism (*p* = 0.034) in SPS patients when compared with the control group.

This might indicate that a diagnostic approach using genetic analysis of the presence of particular SNPs can predict clinically manifesting pregnancy complications developed on the basis of thrombotic events in uteroplacental circulation.

We are self-critically aware of the several limitations of our study—the fact that non-pregnant women were used as the control group, and the limited number of pregnant patients included because of health or personal issues. However, we will continue including patients to our study to contribute to improved knowledge in this field of research. Nevertheless, our study might be regarded as unique because, to the best of our knowledge, only our work has performed a genetic analysis of these selected polymorphisms associated with pregnancy complications in the specific population of at-risk pregnant women with SPS.

To conclude, we sincerely hope that our study might be useful and enrich the general knowledge around sticky platelet syndrome, helping in the management of at-risk pregnant women with SPS.

## Figures and Tables

**Table 1:** Diagnostic criteria of SPS.

	Platelet Aggregation after the Addition of					
	ADP			EPI		
Concentration (μM)	0.58	1.17	2.34	0.55	1.1	11
Reference range of aggregation (%)	0–12	2–36	7.5–55	9–20	15–27	39–80

Legend: ADP—adenosine diphosphate, EPI—epinephrine, SPS—sticky platelet syndrome.

**Table 2:** Contingency table showing the frequency of genotypes of VEGFA rs2010963 in the studied population.

Group		Rs2010963			Total
		CC	CG	GG	
A	Observed	4	22	27	53
	% within row	7.5%	41.5%	50.9%	100.0%
C	Observed	4	23	31	58
	% within row	6.9%	39.7%	53.4%	100.0%
S	Observed	4	20	29	53
	% within row	7.5%	37.7%	54.7%	100.0%
T	Observed	1	9	11	21
	% within row	4.8%	42.9%	52.4%	100.0%
Total	Observed	13	74	98	185
	% within row	7.0%	40.0%	53.0%	100.0%

Legend: group A—recurrent spontaneous abortions, group C—controls, group S—sticky platelet syndrome, group T—thromboembolism, VEGFA—vascular endothelial growth factor A.

**Table 3:** Contingency table showing the frequency of genotypes of *VEGFA* rs3025039 in the studied population.

Group		rs3025039			
		CC	CT	TT	Total
A	Observed	37	15	1	53
	% within row	69.8%	28.3%	1.9%	100.0%
C	Observed	39	17	2	58
	% within row	67.2%	29.3%	3.4%	100.0%
S	Observed	41	12	0	53
	% within row	77.4%	22.6%	0.0%	100.0%
T	Observed	14	6	1	21
	% within row	66.7%	28.6%	4.8%	100.0%
Total	Observed	131	50	4	185
	% within row	70.8%	27.0%	2.2%	100.0%

Legend: group A—recurrent spontaneous abortions, group C—controls, group S—sticky platelet syndrome, group T—thromboembolism, VEGFA—vascular endothelial growth factor A.

**Table 4:** Contingency table showing the frequency of genotypes of *ALPP* rs13026692 in the studied population.

Group		rs13026692			
		AA	AT	TT	Total
A	Observed	27	21	5	53
	% within row	50.9%	39.6%	9.4%	100.0%
C	Observed	27	23	8	58
	% within row	46.6%	39.7%	13.8%	100.0%
S	Observed	23	28	2	53
	% within row	43.4%	52.8%	3.8%	100.0%
T	Observed	6	14	1	21
	% within row	28.6%	66.7%	4.8%	100.0%
Total	Observed	83	86	16	185
	% within row	44.9%	46.5%	8.6%	100.0%

Legend: *ALPP*—alkaline phosphatase, placental, group A—recurrent spontaneous abortions, group C—controls, group S—sticky platelet syndrome, group T—thromboembolism.

**Table 5:** Contingency table showing the frequency of genotypes of *FOX3* rs3761548 in the studied population.

Group		rs3761548			
		GG	TG	TT	Total
A	Observed	20	23	10	53
	% within row	37.7%	43.4%	18.9%	100.0%
C	Observed	21	29	8	58
	% within row	36.2%	50.0%	13.8%	100.0%
S	Observed	14	24	15	53
	% within row	26.4%	45.3%	28.3%	100.0%
T	Observed	9	11	1	21
	% within row	42.9%	52.4%	4.8%	100.0%
Total	Observed	64	87	34	185
	% within row	34.6%	47.0%	18.4%	100.0%

Legend: FOX 3—fork head box protein 3, group A—recurrent spontaneous abortions, group C—controls, group S—sticky platelet syndrome, group T—thromboembolism.

**Table 6:** Multinomial logistic regression—group vs. SNPs.

							95% Confidence Interval	
Group	Predictor	Estimate	SE	Z	*p*	Odds Ratio	Lower	Upper
A-C	Intercept	−0.593	1.575	−0.377	0.707	0.5527	0.0252	12.11
	rs2010963:							
	CG–CC	−0.159	0.785	−0.202	0.84	0.8531	0.1832	3.972
	GG–CC	−0.271	0.774	−0.350	0.727	0.7629	0.1673	3.479
	rs3025039:							
	CC–TT	0.657	1.287	0.51	0.61	1.929	0.1548	24.039
	CT–TT	0.557	1.306	0.427	0.669	1.7459	0.1351	22.556
	rs13026692:							
	AA–TT	0.464	0.642	0.722	0.47	1.5903	0.4517	5.599
	AT–TT	0.405	0.655	0.618	0.536	1.4993	0.4154	5.411
	rs3761548:							
	GG–TT	−0.272	0.587	−0.464	0.643	0.7617	0.2413	2.405
	TG–TT	−0.404	0.563	−0.718	0.473	0.6675	0.2215	2.011
S-C	Intercept	−14.105	0.765	−18.446	<0.001	7.49 × 10^−7^	1.67 × 10^−7^	3.35 × 10^−6^
	rs2010963:							
	CG–CC	−0.347	0.82	−0.424	0.672	0.7065	0.1417	3.522
	GG–CC	−0.382	0.803	−0.475	0.635	0.6827	0.1414	3.296
	rs3025039:							
	CC–TT	13.961	0.422	33.1	<0.001	1.16 × 10^6^	506,157.2	2.64 × 10^6^
	CT–TT	13.383	0.469	28.544	<0.001	648,812	258,843.3	1.63 × 10^6^
	rs13026692:							
	AA–TT	1.224	0.859	1.424	0.155	3.3993	0.6306	18.323
	AT–TT	1.819	0.86	2.116	0.034	6.1658	1.1435	33.246
	rs3761548:							
	GG–TT	−1.320	0.594	−2.223	0.026	0.2671	0.0834	0.856
	TG–TT	−0.847	0.54	−1.568	0.117	0.4286	0.1486	1.236
T-C	Intercept	−3.057	2.239	−1.365	0.172	0.047	5.84 × 10^−4^	3.79
	rs2010963:							
	CG–CC	0.31	1.208	0.257	0.798	1.3633	0.1277	14.555
	GG–CC	0.293	1.197	0.244	0.807	1.34	0.1282	14.005
	rs3025039:							
	CC–TT	−0.205	1.338	−0.154	0.878	0.8143	0.0591	11.211
	CT–TT	−0.169	1.372	−0.123	0.902	0.8448	0.0574	12.434
	rs13026692:							
	AA–TT	0.611	1.163	0.525	0.599	1.8418	0.1886	17.988
	AT–TT	1.536	1.123	1.367	0.172	4.6452	0.5139	41.99
	rs3761548:							
	GG–TT	0.979	1.16	0.844	0.399	2.6613	0.2738	25.869
	TG–TT	0.973	1.138	0.855	0.393	2.6457	0.2841	24.634

Legend: group A—recurrent spontaneous abortions, group C—controls, group S—sticky platelet syndrome, group T—thromboembolism, *p*—*p* value, SE—standard error, SNP—single nucleotide polymorphism, Z—Z-score.

**Table 7:** Post hoc comparisons—rs3025039.

Response Groups	Comparison		Difference	SE	z	*p*	*p*Bonferroni	*p*holm
	rs3025039	rs3025039						
A	CC	CT	−0.02288	0.0794	−0.2881	0.775	1	1
	CC	TT	0.00906	0.246	0.0368	0.971	1	1
	CT	TT	0.03195	0.2503	0.1277	0.899	1	1
C	CC	CT	−0.05383	0.0793	−0.6788	0.503	1	1
	CC	TT	−0.23849	0.2587	−0.9219	0.365	1	1
	CT	TT	−0.18466	0.2625	−0.7035	0.488	1	1
S	CC	CT	0.09291	0.0655	1.4191	0.167	0.502	0.167
	CC	TT	0.31276	0.0585	5.3437	<0.001	<0.001	<0.001
	CT	TT	0.21986	0.0654	3.3641	0.002	0.007	0.005
T	CC	CT	−0.01620	0.0392	−0.4135	0.683	1	1
	CC	TT	−0.08334	0.1501	−0.5552	0.583	1	1
	CT	TT	−0.06715	0.1517	−0.4427	0.661	1	1

Legend: group A—recurrent spontaneous abortions, group C—controls, group S—sticky platelet syndrome, group T—thromboembolism, *p*—*p* value, SE—standard error, SNP—single nucleotide polymorphism, Z—Z-score.

**Table 8:** Post hoc comparisons—rs13026692.

Response Groups	rs13026692	rs13026692	Difference	SE	z	*p*	*p*Bonferroni	*p*holm
A	AA	AT	0.0824	0.075	1.099	0.282	0.845	0.845
	AA	TT	0.0313	0.1276	0.245	0.808	1	1
	AT	TT	−0.0511	0.1271	−0.402	0.691	1	1
C	AA	AT	0.0728	0.0789	0.923	0.364	1	0.54
	AA	TT	−0.1507	0.1338	−1.126	0.27	0.81	0.54
	AT	TT	−0.2235	0.1348	−1.658	0.109	0.327	0.327
S	AA	AT	−0.0695	0.0488	−1.424	0.166	0.497	0.309
	AA	TT	0.0976	0.0666	1.465	0.155	0.464	0.309
	AT	TT	0.1671	0.0688	2.43	0.022	0.066	0.066
T	AA	AT	−0.0857	0.0618	−1.385	0.177	0.532	0.532
	AA	TT	0.0218	0.0675	0.323	0.749	1	0.749
	AT	TT	0.1075	0.0835	1.287	0.209	0.627	0.532

Legend: group A—recurrent spontaneous abortions, group C—controls, group S—sticky platelet syndrome, group T—thromboembolism, *p*—*p* value, SE—standard error, SNP—single nucleotide polymorphism, Z—Z-score.

**Table 9:** Post hoc comparisons—rs3761548.

Response Groups	rs3761548	rs3761548	Difference	SE	z	*p*	*p*Bonferroni	*p*holm
A	GG	TG	0.0457	0.0793	0.5765	0.569	1	1
	GG	TT	−0.00977	0.1095	−0.0892	0.93	1	1
	TG	TT	−0.05547	0.1055	−0.5259	0.603	1	1
C	GG	TG	0.004	0.086	0.0466	0.963	1	1
	GG	TT	0.06965	0.1155	0.6032	0.551	1	1
	TG	TT	0.06565	0.1097	0.5986	0.554	1	1
S	GG	TG	−0.05293	0.0395	−1.3417	0.191	0.573	0.229
	GG	TT	−0.15178	0.0618	−2.4573	0.021	0.062	0.062
	TG	TT	−0.09885	0.0606	−1.6315	0.114	0.343	0.229
T	GG	TG	0.00322	0.0559	0.0577	0.954	1	0.954
	GG	TT	0.09189	0.0714	1.2866	0.209	0.628	0.503
	TG	TT	0.08867	0.0625	1.4181	0.168	0.503	0.503

Legend: group A—recurrent spontaneous abortions, group C—controls, group S—sticky platelet syndrome, group T—thromboembolism, *p*—*p* value, SE—standard error, SNP—single nucleotide polymorphism, Z—Z-score.

**Table 10:** Model results of log likelihood ratio tests.

	X^2^	df	*p*
rs2010963	1.22835	2	0.541
rs3025039	0.39821	1	0.528
rs13026692	1.50814	2	0.47
rs3761548	3.13461	2	0.209
age	0.12124	1	0.728
ASA and LMWH	29.34294	4	<0.001
other thrombophilia	0.00841	1	0.927

Legend: ASA—acetylsalicylic acid, df—degrees of freedom, LMWH—low molecular weight heparin, *p*—*p* value, X^2^—Chi-squared test.

**Table 11:** Post hoc comparisons—rs2010963.

Comparison							
rs2010963	rs2010963	exp (B)	SE	z	*p*	*p*Bonferroni	*p*holm
CC	CG	0.217	0.482	−0.689	0.491	1.000	0.982
CC	GG	0.652	1.296	−0.215	0.830	1.000	0.982
CG	GG	3.001	3.134	1.053	0.293	0.878	0.878

Legend: exp(B)—exponential value of B, *p*—*p* value, SE—standard error, z—Z-score.

**Table 12:** Post hoc comparisons—rs3025039.

Comparison							
rs3025039	rs3025039	exp (B)	SE	z	*p*	*p*Bonferroni	*p*holm
CC	CT	2.02	2.32	0.613	0.540	0.540	0.540

Legend: exp(B)—exponential value of B, *p*—*p* value, SE—standard error, z—Z-score.

**Table 13:** Post hoc comparisons—rs13026692.

Comparison							
rs13026692	rs13026692	exp (B)	SE	z	*p*	*p*Bonferroni	*p*holm
AA	AT	1.19	1.17	0.17855	0.858	1.000	1.000
AA	TT	5.84 × 10^8^	7.25 × 10^12^	0.00163	0.999	1.000	1.000
AT	TT	4.90 × 10^8^	6.08 × 10^12^	0.00161	0.999	1.000	1.000

Legend: exp(B)—exponential value of B, *p*—*p* value, SE—standard error, z—Z-score.

**Table 14:** Post hoc comparisons—rs3761548.

Comparison							
rs3761548	rs3761548	exp (B)	SE	z	*p*	*p*Bonferroni	*p*holm
GG	TG	6.96	9.17	1.473	0.141	0.422	0.341
GG	TT	10.66	15.95	1.582	0.114	0.341	0.341
TG	TT	1.53	1.94	0.337	0.736	1.000	0.736

Legend: exp(B)—exponential value of B, *p*—*p* value, SE—standard error, z—Z-score.

**Table 15:** Post hoc comparisons—ASA_LMWH.

Comparison							
ASA_LMWH	ASA_LMWH	exp(B)	SE	z	*p*	*p*Bonferroni	*p*holm
ASA	ASA	3.14 × 10^8^	3.07 × 10^12^	0.00200	0.998	1.000	1.000
ASA	ASA, LMWH	1.01 × 10^9^	4.30 × 10^12^	0.00487	0.996	1.000	1.000
ASA	LMWH	0.344	0.334	−1.10018	0.271	1.000	1.000
ASA	LMWH a ASA	5.04 × 10^−10^	3.16 × 10^−6^	−0.00341	0.997	1.000	1.000
ASA	ASA, LMWH	3.223	34367.226	1.10 × 10^−4^	1.000	1.000	1.000
ASA	LMWH	1.10 × 10^−9^	1.07× 10^−5^	−0.00211	0.998	1.000	1.000
ASA	LMWH a ASA	2.22 × 10^−16^	2.58 × 10^−12^	−0.00353	0.997	1.000	1.000
ASA, LMWH	LMWH	3.40 × 10^−10^	1.45 × 10^−6^	−0.00512	0.996	1.000	1.000
ASA, LMWH	LMWH a ASA	2.22 × 10^−16^	1.68 × 10^−12^	−0.00555	0.996	1.000	1.000
LMWH	LMWH a ASA	1.46 × 10^9^	9.20 × 10^−6^	−0.00324	0.997	1.000	1.000

**Table 16:** Post hoc comparisons—other thrombophilia.

Comparison							
Other_Thrombophilia	Other_Thrombophilia	exp(B)	SE	z	*p*	*p*Bonferroni	*p*holm
no	yes	0.898	1.06	−0.0915	0.927	0.927	0.927

Legend: exp(B)—exponential value of B, *p*—*p* value, SE—standard error, z—Z-score.

## Data Availability

Not applicable.
